# The goal of geroscience is life extension

**DOI:** 10.18632/oncotarget.27882

**Published:** 2021-02-02

**Authors:** Mikhail V. Blagosklonny

**Affiliations:** ^1^Roswell Park Cancer Institute, Buffalo, NY 14263, USA

**Keywords:** aging, longevity, rapamycin, mTOR, metformin

## Abstract

Although numerous drugs seemingly extend healthspan in mice, only a few extend lifespan in mice and only one does it consistently. Some of them, alone or in combination, can be used in humans, without further clinical trials.

## INTRODUCTION

“Although we do not know everything about aging, we now know enough to start its pharmacologic suppression using clinically approved drugs.” Published in 2010, these opening words of the paper entitled “Increasing healthy lifespan by suppressing aging in our lifetime: preliminary proposal” are still relevant today [1]. The proposal was based on hyperfunction theory that aging is a continuation of development, driven in part by growth-promoting pathways, such as mTOR [2]. Hyperfunction (inappropriate activation) of these signaling pathways directly drive all age-related diseases, which are manifestations of aging. We just need clinically available inhibitors (drugs) of these signaling pathways to extend both healthspan and lifespan, by slowing aging.

Here I discuss a practical approach as to how to make the current generation of adults live longer and healthier, without conducting lifelong randomized clinical trials. Even if such trials would be started, only the next generation may benefit. But such clinical trials are not needed anymore. For practical purposes, it is both necessary and sufficient that a drug consistently and significantly extend lifespan in mice (and other mammals, if tested) and be already approved for any indication in humans. Given that no proof of life extension in humans (and other long-lived mammals) can be available in our lifetime, we need strong evidence in rodents. I will discuss that the requirement of life extension must be strict. A mere extension of healthspan is not enough: drugs that fail to extend lifespan in mice will fail to extend lifespan in humans, if used as a monotherapy. Yet, in rational combinations with life-extending drugs, “healthspan-only” drugs may extend lifespan further. Here I will review drugs that extend lifespan and healthspan in mammals (e.g., rapamycin), in contrast to those that may affect only healthspan without lifespan (e.g., resveratrol), and discuss how to proceed with clinical application of lifespan-extending drugs.

### Failure of anti-oxidants

The goal of longevity research is life extension. Instead, most studies are focused on life-shortening. Many things can shorten lifespan without causing normal aging. For example, car accidents shorten lifespan, but they do not cause aging. Knockout of antioxidant enzymes can shorten lifespan. But antioxidants do not extend lifespan [3–12]. Furthermore, antioxidants increase mortality in humans [13–17]. Clinical trials of antioxidants have been terminated because of increased cancer incidence [13–17]. In agreement, antioxidants promote cancer in mice [18, 19]. Yet, thousands of publications describe mechanisms of life extension by the exact same antioxidants that do not extend lifespan. To study mechanisms of life extension, there must be life extension at least.

Certainly, reactive oxygen species (ROS) cause molecular damage, and this damage would eventually kill the organism. But no organism lives long enough to die from accumulation of molecular damage because hyperfunctional (in part, mTOR-driven) aging terminates life first [9]. If artificially accelerated by knocking out genes, molecular damage may become life limiting. But in normal animals and humans, it is not. We cannot extend life by targeting a non-life-limiting process.

### The goal of geroscience

The goal of geroscience is extension of lifespan by extending healthspan. Standard medical interventions can prolong lifespan without extending healthspan (e.g., using a ventilator in comatose patient) but anti-aging interventions increase lifespan by slowing aging and thus delaying age-related diseases (extending healthspan). One of the notions of hyperfunction theory is that age-related diseases can be prevented and treated by slowing down aging with anti-aging drugs, such as rapamycin, as discussed in 2006–2012 [2, 20–22] and later named the geroscience hypothesis [23–26].

### Lifespan vs healthspan

Healthspan is a period of life without age-related diseases [27]. It is disease-free survival. (Note: A patient may view healthspan as a symptom-free survival, a subjective well-being, but this definition is not used in animal studies.) Since animals and humans die from age-related diseases, an increase in healthspan is expected to increase lifespan. For example, in centenarians, healthspan is increased (this is why they are centenarians) [28]. Calorie restriction (CR) delays diseases and thus extends lifespan in rodents [29]. So healthspan is a surrogate for lifespan.

**Figure 1:**
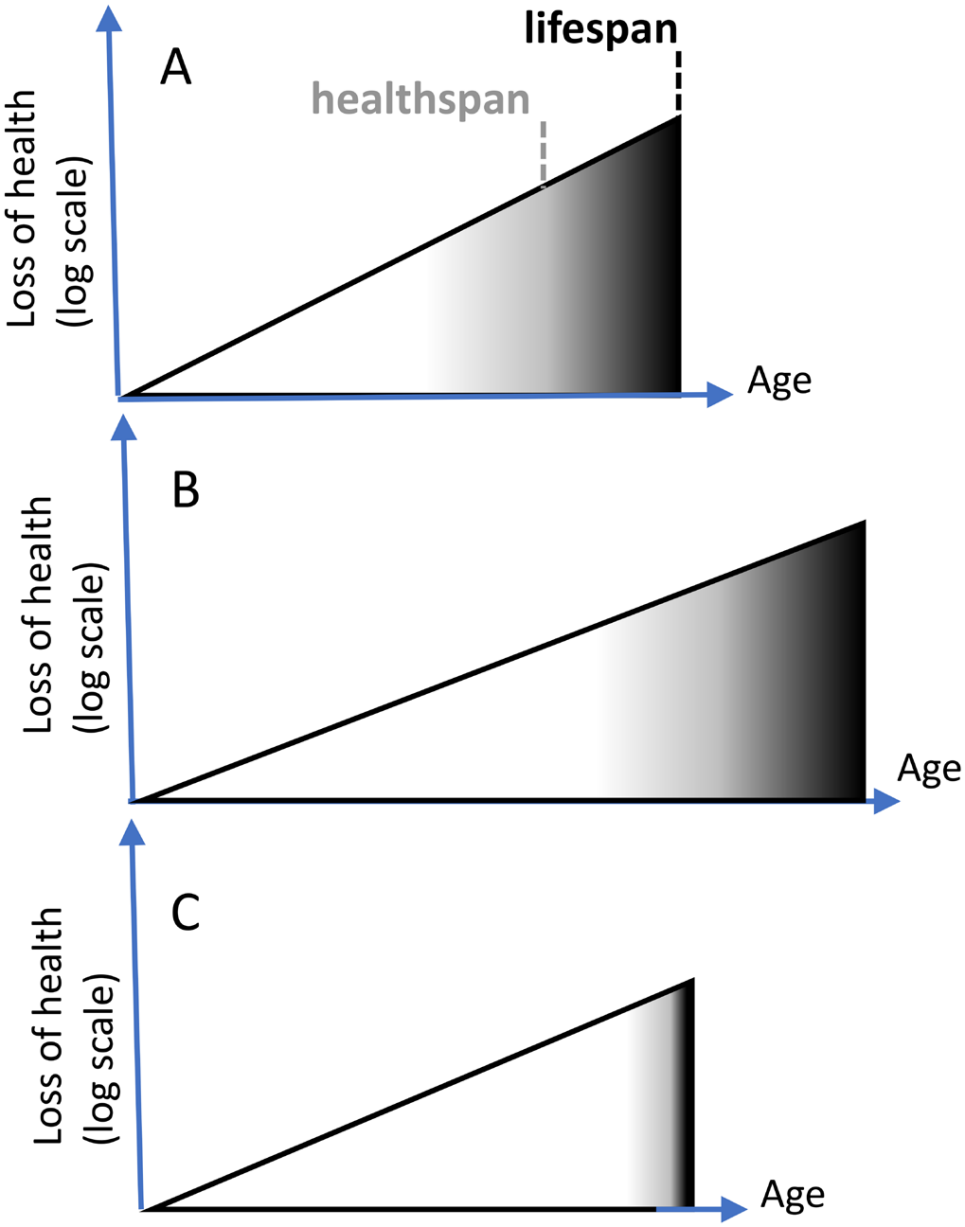
Extension of healthspan extends lifespan. (**A**) Healthspan is the period of life without diseases. Diseases (black color) terminate lifespan. Subclinical aging (white color) progresses to pre-diseases (gray) and diseases (black). X axis: age. Y axis: loss of health (a sum of diseases) in log scale. (**B**) Longevity intervention slows aging and extends healthspan, automatically extending lifespan. (**C**) Unrealistic scenario “Healthspan without lifespan”. Compressed morbidity (black). Either diseases progress instantly, or animals die from “health” rather than from diseases.

However, in comparison with lifespan, healthspan is difficult to measure, especially in animals [27]. The end of healthspan is subjective, because pre-diseases progress slowly towards diseases (Figure 1A: gray zone). Besides, it’s not one disease but the sum of all diseases. In mice, diseases are difficult to diagnose until animal death. Given this, why not then measure healthspan and lifespan simultaneously?

So why has healthspan become so popular in animal studies? The reason is that only a few drugs were shown to extend lifespan in mammals. Other drugs seemingly increase healthspan but do not extend lifespan. This is considered an acceptable and even desirable effect [27]. But it is not. Increased healthspan must automatically increase lifespan (Figure 1A and 1B), if healthspan represents good health. Animals, including humans, do not die from good health, they die from age-related diseases. If diseases are delayed, an animal will live longer. (Symptomatic treatment (e.g., opioids for pain) and placebo may prolong subjective well-being of humans without extending lifespan, but such treatments are not a subject of geroscience).

Consider a scenario in which lifespan is not increased, while healthspan is increased. To keep lifespan constant, while increasing healthspan, diseases must be compressed (Figure 1C): start later but kill faster. For example, in this scenario, cancer kills an organism in a matter of minutes, instead of months. This is impossible (Note: One apparent exception is the illusion of disease compression in centenarians due to poor diagnostics and lack of medical treatment at the oldest age, as discussed later). Otherwise, an increase of healthspan must increase at least median lifespan (Figure 1B).

So how is it possible that some senolytics, NAD boosters and resveratrol, increase healthspan without lifespan? The simplest explanation is that they do not increase healthspan at all, because such studies use irrelevant or ambiguous markers of health. Ambiguous parameters can be associated with either good or bad health, depending on the underlying cause. For example, similar changes in insulin signaling are associated with either slow or fast aging, depending on the mTOR activity [30].

The second explanation is that some diseases are not deadly, so that their treatment does not extend lifespan but improve quality of life. Yet, healthspan should not be measured by non-deadly diseases only. After all, aging is an exponential increase of death with age and should be measured by deadly diseases.

Third, some age-dependent alterations are not life-limiting, simply because hyperfunctional aging terminates life first. For example, accumulation of mitochondrial DNA mutations, telomere shortening, NAD+ depletion do not reach lethal threshold during animal life time (see Blagosklonny MV. When longevity drugs do not increase longevity: Unifying development-driven and damage-induced theories of aging, 2021, in press). So, their improvement is not translated in life extension. They may become life-limiting either when they are accelerated or when normal aging is decelerated. For example, telomerase-knockout mice die young from bone marrow failure. In such mice, testosterone extends lifespan by extending telomeres [31]. But, testosterone does not extend life in normal animals. As a hypothetical possibility, treatment of non-limiting deteriorations with “healthspan” drugs may extend lifespan, when life-limiting aging is sufficiently decelerated and an animal (or human) lives long enough to die from this deterioration. This is an exciting possibility because then “helthspan without lifespan drugs” may increase life even further, as will be discussed in detail (Blagosklonny MV. When longevity drugs do not increase longevity: Unifying development-driven and damage-induced theories of aging, 2021, in press).

### If a drug does not increase lifespan in mammals, there is no reason to think it would do so in humans

Even if a drug DOES increase lifespan in mice and other mammals, gerontologists are still skeptical that it will work in humans. Consider an example. Calorie restriction (CR) extends lifespan in mice, rats and even monkeys [32, 33]. CR must extend lifespan in humans because it delays all age-related diseases in humans [29]. Still it is debated whether it would extend life in humans. Some gerontologists think that it will not [34]. Imagine, if CR would not increase lifespan in any mammal including mice. Would we then think that it may mysteriously extend life in humans? No. But then why are drugs that do not extend life in mice still being considered to extend life in humans. To reasonably expect that a drug will extend lifespan in humans, a drug must extend medium and maximum lifespan in genetically heterogeneous mice, multiple strains of mice, and cancer-prone mice. (Note: Cancer is delayed when aging is slow. For example, cancer is rare in long-lived naked mole rats and centenarians).

### Resveratrol

Three studies in mice failed to show life extension by resveratrol [35–37]. In one of them, resveratrol improved health without extending lifespan [35]. Similarly, pharmacological doses of resveratrol delayed vascular aging but did not extend lifespan in Wistar rats [38]. In 2006 it was shown that resveratrol increased mean lifespan in mice on a high-calorie diet [39]. A high-calorie diet (HCD) shortened mean lifespan by increasing early death, and resveratrol prevented that shortening but did not extend lifespan beyond the control standard diet [39]. So, resveratrol may reverse life-shortening caused by HCD, but does not extend normal lifespan.

### Rapamycin

Since 2009, dozens of studies showed that rapamycin extends medium and maximum lifespan in both males and females in all strains of normal mice tested as well as in some cancer-prone and short-lived mice [36, 40–70]. Rapamycin extended life when was given at old or young age, constantly with food, intermittently, or even transiently. In some short-lived mutant mice, rapamycin more than doubled lifespan [50, 54, 60, 61, 64]. The higher the dose, the longer lifespan [57, 61, 70]. In one exception that emphasizes the rule, rapamycin slightly shortened lifespan in artificial mice lacking telomerase that failed to grow and die young but not from aging [68]. As predicted by theory [2], rapamycin, which slows growth and cell proliferation, will be unfavorable during developmental growth in mice that fail to grow. mTOR drives growth early in life and aging (a continuation of growth) later in life. In fact, in the same study, rapamycin dramatically extended lifespan in normal parental mice [68].

### Curcumin

Curcumin, fed to mice beginning at 12 months of age, did not extend lifespan in male F1 hybrid mice [71]. Also, in genetically heterogeneous mice, curcumin administered beginning at 4 months of age had no effect on lifespan of male or female mice [37]. Thus, curcumin never was shown to extend lifespan in any mammals.

### Quercetin

As shown in 1982, dietary supplement of 0.1% quercetin significantly decreased lifespan of mice [72]. This disappointing result is still non-disputed. In a second study, quercetin, fed beginning at 12 months of age, did not extend lifespan in male F1 hybrid mice [71]. However, a senolytic combination of quercetin with dasatinib (Q + D combination) increased median lifespan by 6.3% (Figure 6I in reference [73]) in C57BL/6 mice [73]. Q alone was not tested in this study [73].

(Note: Instead of medium lifespan, the abstract lists post-treatment survival, increased by 36%.) The treatment was started at age 24–27 months and administered every 2 weeks by oral gavage for 3 consecutive days.

### Spermidine

Spermidine is an abundant natural polyamine contained in all organisms and human food. Spermidine did not extend median and maximum lifespan of the middle-aged male Sprague-Dawley rats (although spermidine increased “healthspan”) [74]. In C57BL/6J wild-type female mice, spermidine increased medium (but not maximum) lifespan by approximately 10% when given either life-long or late-life [75].

### NAD boosters

Nicotinamide riboside (NR), a NAD+ booster, extends lifespan in mice by 4.7% (marginally statistically significant *P* = 0.034) [76]. This modest result is the best so far. In another study, nicotinamide (NAM), at low and high doses, did not extend lifespan in male C57BL/6J mice on standard and high-fat diets [77]. Noteworthy, NAD boosters dramatically increase lifespan in short-lived mice with some progeroid syndromes [78–80]. However, these mice die young not from normal aging but from pathologies such as liver fibrosis and bone marrow failure [78]. In contrast, there was no benefit of NR or NMN in normal mice [78, 80].

### Berberine

In one study, berberine extended lifespan in C57BL/6J male mice [81]. Of note, berberine inhibits the mTOR pathway in cell culture [82].

### Fisetin

In old mice, fisetin slightly extended lifespan, but only a few mice were used in the study (Figure 5A in reference [83]).

### Metformin

The effect of metformin on lifespan in mice depends on strain, age, sex and other conditions. For example, in inbred 129/Sv mice, metformin (100 mg/kg in drinking water) decreased the mean lifespan of male mice (by 13.4%) and increased the mean lifespan of female mice (by 4.4%) [84]. In female SHR mice, metformin increased mean lifespan by 37.8%, and maximum life span by 10.3% [85]. If started early in life, metformin increased lifespan in female SHR mice [86]. In another study, lifespan was not affected by metformin alone but was decreased by a combination of metformin with SRT1720, a sirtuin1-activator [87]. In 129/Sv mice, neonatal metformin treatment at the 3rd, 5th and 7th days after birth extended lifespan in male, but not in female, mice [88].

A famous study entitled “Metformin improves healthspan and lifespan in mice” actually shows that “low” dose of metformin (0.1% w/w in diet) slightly extended lifespan in C57BL/6 male mice, while a higher dose (1% w/w in diet) considerably decreased lifespan [89]. In genetically heterogeneous mice, however, metformin (0.1% w/w in diet) did not extend lifespan [90]. In this study, metformin combined with rapamycin (14 ppm) robustly extended lifespan, but treatment with rapamycin alone was not tested in the same study [90]. In two studies, metformin increased lifespan in cancer-prone HER-2/neu transgenic mice [91, 92]. In conclusion, metformin consistently increases lifespan in short-lived, cancer-prone mice. In normal mice, effects of metformin are inconsistent, ranging from life extension to life shortening.

### 17-alpha-estradiol

(17aE2) robustly extended both median and maximal lifespan, but only in males [90, 93].

### Acarbose

The alpha-glucosidase inhibitor, acarbose, increased median longevity in males and 90th percentile lifespan in both sexes [90, 93–95]. Acarbose blocks digestion of complex carbohydrates. So, the treatment is an equivalent of carbohydrate-free diets such as ketogenic diets in humans. Low carb-diets may increase lifespan and can be used in combination with rapamycin [96].

### Enalapril

Angiotensin converting enzyme (ACE) inhibitors (ACEi), such as enalapril, lisinopril and ramipril, and angiotensin receptor blockers (ARB) such as losartan, telmisartan and valsartan are widely used to treat hypertension. Both ACE inhibitors and ARBs are commonly used in patients with hypertension, heart failure, coronary artery disease, diabetes and chronic kidney disease (CKD). In 1993, it was shown that enalapril, at various doses, increased survival in mice. The authors speculated that enalapril must decrease oxidative damage, as required by dogma [97]. Losartan and enalapril prolonged lifespan in Wistar rats. All control rats died by the age of 28 months. In the treatment groups, 62% of rats were alive. Mean survival was increased by 21% and 19% for enalapril and losartan respectively (*p* < 0.001) [98]. Enalapril treatment increased lifespan in Wistar rats on standard and palatable hyper lipidic diets [99]. Ramipril (ACEi) in combination (but not alone) with Simvastatin extended lifespan of long-lived, B6C3F1 male mice [100].

### Interpretation of mouse longevity data

Although hundreds of recent reviews proclaim a wide arsenal of “emerging” drugs that “promise” to extend healthspan and lifespan, these drugs either do not extend lifespan in mice, or data is not sufficient. For example, resveratrol does not extend lifespan in mammals in any study [35–38]. In one study, it reverses life-shortening caused by HCD [39]. But reversal of life-shortening does not imply anti-aging activity. (Consider an analogy. In animals dying prematurely from type 1 diabetes, insulin therapy extends lifespan. But insulin cannot extend normal lifespan. Insulin is a pro-aging rather than anti-aging hormone.) Similarly, numerous flavonoids including curcumin and quercetin were not shown to prolong lifespan in mice and rats.

One drug, rapamycin, stands out [101]. Rapamycin extends lifespan in all numerous studies in normal mice and doubles lifespan in several short-lived mice [50, 54, 60, 61, 64]. Unlike other drugs discussed here, rapamycin is a gerostatic, a drug that decelerates cellular conversion to senescence in cell culture [102–104]. It has only one molecular target, the mTOR complex 1. The mTORC1 network is involved in aging and age-related diseases. Cellular senescence and organismal aging are a continuation of growth, which is driven in part by the mTOR pathway [2]. It was predicted that rapamycin must extend lifespan before it was shown in any animal [105].

In comparison, resveratrol has no relevant molecular target at least in mammals, meaning that it has all possible targets, especially at super-physiological concentrations [106], including mTOR at cytotoxic doses [107, 108] and topoisomerase, a target for highly cytotoxic chemotherapy [109]. Similarly, flavonoids and metformin have multiple potential targets.

Metformin extends lifespan in some cancer-prone mice and two normal strains, depending on doses, sex and schedules. Its life-extending effects are limited by toxicity at higher doses that may even shorten lifespan. However, clinical doses of metformin are well-tolerated in humans. Retrospective and prospective data of numerous studies indicate that metformin decreases all-cause mortality in patients taking it for many different diseases [110–112]. It also decreases incidence of cancer, when taking for other indications. Potential benefits of a metformin/rapamycin combination were suggested [113]. Yet, a combination of metformin and rapamycin should be re-tested to include a rapamycin-alone group.

ACE inhibitors (ACEi) decrease all-cause mortality in patients with various diseases [114–116]. Although, many studies found life-extending benefits of ACEi but not ARB, some studies showed equal benefits of ACEi and ARB [117]. Animal studies showed consistent life extension from ACEi alone [97–99] or in combination with statins [100]. Disruption of the Angiotensin II type 1 receptor increases median and maximum lifespan in mice by 26% [118].

A combination of rapamycin, with metformin, aspirin, ACEi and other drugs was discussed in detail [113].

### Gerostatics

Gerostatics are drugs that decelerate conversion to cell senescence. Gerostatics are also cytostatics, drugs that decelerate cell proliferation [119]. In contrast to senolytics, they do not kill senescent or any other types of cells. Gerostatics have been predicted by hyperfunction theory of aging [2]. Rapamycin and other rapalogs (e.g., everolimus), inhibitors of the mTOR kinase (known as pan-mTOR inhibitors), S6K, PI3K, MEK and MDM-2 all target growth- and senescence-promoting networks and decelerate cellular senescence in cell culture [102–104, 120–122]. Although their gerostatic properties were described more than decade ago, they have not been tested on life extension in mammals. A MEK inhibitor is a clinically available drug and extends lifespan in Drosophila [123], but its effects on longevity were not tested in mice.

I expect that a combination of low doses of pan-mTOR and MEK inhibitors with high doses of rapamycin would extend life further compared with rapamycin alone. That could be the next important advance in the anti-aging field since the discovery of anti-aging properties of rapamycin.

### Surrogate lifespan trials in humans

In humans, it is nearly impossible to conduct life-long clinical trials to measure lifespan. In surrogate trials, anti-aging drugs can be validated by treating and preventing age-related diseases [21]. Aging is a sum of all age-related diseases, which terminate life. If all diseases are delayed, then lifespan must increase. In other words, if healthspan is increased, lifespan is increased automatically. Anti-aging drugs such as rapamycin are more effective for disease prevention than for their treatment [2], as emphasized for cancer prevention [20]. While treating one age-related disease, an anti-aging drug will delay progression of other diseases (e.g., cancer and Alzheimer’s disease) and syndromes (e.g., frailty) and cosmetic appearance (e.g., grey hair and winkled skin) [21].

In humans, lifespan can be increased without increasing healthspan by standard medical interventions [124]. In a comatose patient after stroke, a ventilator extends lifespan without increasing healthspan. For example, defibrillation may resurrect a patient, extending lifespan by decades without affecting healthspan. Anti-aging interventions increase lifespan, primarily by increasing healthspan. For example, if stroke is delayed, lifespan will be increased. An increased healthspan will not compress morbidity, as erroneously suggested by some gerontologists. (Note: compression of morbidity can only be achieved by denying the very elderly the best medical care, which otherwise expands the morbidity phase in lesser elderly people. This is observed in centenarians who are not even diagnosed with diseases, even less treated.) The greatest achievement of modern medicine is dramatic extension of lifespan by expanding the morbidity phase, not by compressing it.

Anti-aging drugs are expected primarily work by expanding healthspan [124, 125]. But the morbidity period will be extended (decompressed) too, because disease progression will be decelerated.

### Validation of anti-aging drugs and TAME

Based on the notion that aging is the sum of all age-related diseases, it was proposed to validate anti-aging drugs by treating age-related diseases. Published in 2009, the abstract of an article entitled “Validation of anti-aging drugs by treating age-related diseases” stated: “Humans die from age-related diseases, which are deadly manifestations of the aging process. In order to extend lifespan, an anti-aging drug must delay age-related diseases. Once a drug is used for treatment of any one chronic disease, its effect against other diseases (atherosclerosis, cancer, prostate enlargement, osteoporosis, insulin resistance, Alzheimer’s and Parkinson’s diseases, age-related macular degeneration) may be evaluated in the same group of patients. If the group is large, then the anti-aging effect could be validated in a couple of years.” [21]. So, anti-aging drugs were suggested to be validated in patients with diseases, rather than in healthy individuals [21]. Specifically, it was discussed for metformin and rapamycin [21]. This proposal has been implemented by Nir Barzilai and others in a placebo-controlled, randomized, double-blind, clinical trial the “Targeting Aging with Metformin” or “TAME”, which enrolls patients with any age-related disease to determine whether metformin is effective at delaying the incidence of a composite of multiple age-related diseases, geriatric syndromes and functional health [126].

### The origin of geroscience

By 2006, clinical data became available for retrospective analysis of effects of metformin and rapamycin on several age-related diseases [2, 21]. The concept of treatment of age-related diseases was discussed in “Prevention of cancer by inhibiting aging” [20] and “Prospective treatment of age-related diseases by slowing down aging” [22]. The concept of treatment of age-related diseases by slowing down aging [22] was later named the geroscience hypothesis [23, 24, 25, 26]. The geroscience hypothesis, however, is incomplete, because it assumes that aging is caused by molecular damage. Aging is obligatory, whereas diseases are not [127]. So, the mechanistic link remains elusive. According to the geroscience hypothesis, aging is a risk factor for diseases [127]. According to hyperfunction theory, in contrast, aging is a sum of all age-related diseases, not their risk factors. The molecular basis of aging is hyperfunction (unnecessary activation) of signaling pathways, such as mTOR, which drive diseases directly. Aging and diseases are two sides of the same coin: diseases are manifestations of aging.

### Senolytics

By the strict definition proposed by Kirkland and Tchkonia, senolytics should prolong life specifically by killing senescent cells, not by off-target mechanisms [128]. But strictly speaking, such drugs do not exist yet [Note: As an example of off-target mechanisms, at high doses, some “senolytics” inhibit mTOR.]

Although a wide variety of compounds are called senolytics, most of them do not extend lifespan by any mechanism in any mammal. Currently, only two senolytics, namely fisetin [83] and a combination of quercetin with dasatinib (Q + D combination) [73] showed life extension in mice. At the time of writing, life extension was shown in just one study for each of them, though, the fisetin-treated group included just a few mice [83]. However, even these modest results are important, because these drugs are clinically available. Especially, fisetin and quercetin are available as supplements and have already been safely taken by millions of people before the term senolytic was even coined. Dasatinib is a prescription drug approved in 2006 and is safe for human use. A combination of Q + D was tested in humans and, in doses that decrease numbers of senescent cells, they were safe with minimal side effects [129].

### Life extension in mammals and safety in humans

Kirkland and Tchkonia, who have developed these senolytics, warned: “Unless and until such clinical trials are completed and demonstrate safety, tolerability, target engagement and effectiveness, candidate senolytics should not be prescribed or used by general patient populations. They should only be administered in the course of carefully monitored clinical trials.” [130].

Although well intended, this warning is unjustified. Fisetin and quercetin have already been sold as supplements for many years. Why should patients stop taking them? Just because F and D were recently shown to prolong life and health in mice? In Kirkland *et al*., outstanding work, fisetin and a combination of Q plus D prolonged life in mice, not shortened life. Why then should the entire generation of the elderly stop using fisetin, and Q plus D until clinical trials prove that they extend life in humans, and, even more, by a specific mechanism?

The same is applicable to any other drugs that have been used in humans before any studies showed life-extension in animals: rapamycin, metformin and ACEi.

Ironically, some mouse research warns of the metabolic dangers of rapamycin and a ketogenic diet, even though these treatments have been safely used in humans for decades. But these metabolic effects are life-extending in mice, which live longer and healthier. In any case, if data in animals contradict human data, it means that an animal model is irrelevant. Animal models are most needed in the absence of human data. Thus, it is lifespan data that is crucial in mice, because it is unavailable in humans. In humans, the proof of life extension is practically impossible in a short term. Even life extension by calorie restriction (and any diet) was not proven (or disproven) in humans despite decades of research. In forthcoming clinical trials, we cannot expect proof of life extension, only improved health, at doses that do not cause side effects in humans. (Note: Individual anti-aging doses of rapamycin are side-effect-free by definition: the highest dose that is tolerated by a patient). Healthspan and side-effects in humans. Lifespan in mice. Not vice versa.

### How to proceed

We have one life to live and cannot wait for results in others, if we want to live longer ourselves. Although placebo-controlled, randomized, life-long clinical trials should be started, we may only know the answer in a few decades. However, such trials will not even be started, at least not soon. But, fortunately, they are not needed.

Some life-extending drugs are already approved for human use: supplements (fisetin, vitamin B3 and its analogs), over-the-counter medicine (aspirin) and prescription drugs (rapamycin, metformin, dasatinib, enilopril). Do we need to wait for results of future clinical trials or must we simply follow the law? Prescription drugs are allowed by law to be prescribed and used under doctor supervision. That is so simple.

The law is already strict towards rapamycin and metformin, by requiring prescription. In comparison, alcohol and tobacco do not require prescription and doctor supervision. Smoking has no health benefit and dramatically shortens lifespan, accelerating all diseases. While smoking causes cancer, rapamycin prevents it, including smoke-caused lung cancer. Is it then paradoxical that alcohol and tobacco are sold without prescriptions, whereas rapamycin and metformin are not.

Metformin and rapamycin are FDA-approved prescription drugs that are safe by FDA definition. All necessary clinical trials for safety were conducted more than 20 years ago, and then these drugs were used by millions. Hundreds of new trials were conducted later because applications of rapamycin and its analog everolimus were constantly extending. Now is time for longevity clinics. This is the last chance for the current generation to live longer [131].

For example, rapamycin-based personalized treatment can be designed as Phase 1 B clinical trial, with dose escalation to reach dose-limiting side-effects. Then, rapamycin can be used at a lower dose without side effects. Thus, anti-aging treatment with rapamycin can be without side effects by definition. This is important to emphasize. Whatever off-target side effects anti-aging drugs may have, they will be avoided. Treatment is highly individual, because doses of rapamycin must be individual: the highest dose that does not cause side effect in a particular patient. (Note: Blood levels of rapamycin vary widely in different people at the same dose). Such trial-like treatments require no funding, because they can be paid for by participants. If we want to live longer, we have no choice, but to use drugs such as rapamycin, which extends life in short-lived mammals and is approved for humans use. After all, humans are mammals, and there is no reason to think that they will not work in humans.

### Disclaimer

This review is intended for a professional audience. This article does not represent medical advice or recommendations to patients. The media should exercise caution and seek expert medical advice for interpretation, when referring to this article. Medical doctors interested in this topic may e-mail the author at Blagosklonny@rapalogs.com or follow me at Twitter @Blagosklonny.

## References

[1] Blagosklonny MV . Increasing healthy lifespan by suppressing aging in our lifetime: preliminary proposal. Cell Cycle. 2010; 9:4788–94. 10.4161/cc.9.24.14360. 21150328

[2] Blagosklonny MV . Aging and immortality: quasi-programmed senescence and its pharmacologic inhibition. Cell Cycle. 2006; 5:2087–102. 10.4161/cc.5.18.3288. 17012837

[3] Keaney M , Gems D . No increase in lifespan in Caenorhabditis elegans upon treatment with the superoxide dismutase mimetic EUK-8. Free Radic Biol Med. 2003; 34:277–82. 10.1016/s0891-5849(02)01290-x. 12521609

[4] Doonan R , McElwee JJ , Matthijssens F , Walker GA , Houthoofd K , Back P , Matscheski A , Vanfleteren JR , Gems D . Against the oxidative damage theory of aging: superoxide dismutases protect against oxidative stress but have little or no effect on life span in Caenorhabditis elegans. Genes Dev. 2008; 22:3236–41. 10.1101/gad.504808. 19056880 PMC2600764

[5] Perez VI , Bokov A , Van Remmen H , Mele J , Ran Q , Ikeno Y , Richardson A . Is the oxidative stress theory of aging dead? Biochim Biophys Acta. 2009; 1790:1005–14. 10.1016/j.bbagen.2009.06.003. 19524016 PMC2789432

[6] Ristow M , Schmeisser S . Extending life span by increasing oxidative stress. Free Radic Biol Med. 2011; 51:327–36. 10.1016/j.freeradbiomed.2011.05.010. 21619928

[7] Ristow M , Schmeisser K . Mitohormesis: Promoting Health and Lifespan by Increased Levels of Reactive Oxygen Species (ROS). Dose Response. 2014; 12:288–341. 10.2203/dose-response.13-035.ristow. 24910588 PMC4036400

[8] Van Raamsdonk JM , Hekimi S . Superoxide dismutase is dispensable for normal animal lifespan. Proc Natl Acad Sci U S A. 2012; 109:5785–90. 10.1073/pnas.1116158109. 22451939 PMC3326508

[9] Blagosklonny MV . Aging: ROS or TOR. Cell Cycle. 2008; 7:3344–54. 10.4161/cc.7.21.6965. 18971624

[10] Gems D , de la Guardia Y . Alternative Perspectives on Aging in Caenorhabditis elegans: Reactive Oxygen Species or Hyperfunction? Antioxid Redox Signal. 2013; 19:321–9. 10.1089/ars.2012.4840. 22870907 PMC5395017

[11] Selman C , McLaren JS , Collins AR , Duthie GG , Speakman JR . Deleterious consequences of antioxidant supplementation on lifespan in a wild-derived mammal. Biol Lett. 2013; 9:20130432. 10.1098/rsbl.2013.0432. 23825087 PMC3730656

[12] Selman C , McLaren JS , Meyer C , Duncan JS , Redman P , Collins AR , Duthie GG , Speakman JR . Life-long vitamin C supplementation in combination with cold exposure does not affect oxidative damage or lifespan in mice, but decreases expression of antioxidant protection genes. Mech Ageing Dev. 2006; 127:897–904. 10.1016/j.mad.2006.09.008. 17092545

[13] Bjelakovic G , Nikolova D , Gluud LL , Simonetti RG , Gluud C . Mortality in randomized trials of antioxidant supplements for primary and secondary prevention: systematic review and meta-analysis. JAMA. 2007; 297:842–57. 10.1001/jama.297.8.842. 17327526

[14] Bjelakovic G , Nikolova D , Gluud C . Meta-regression analyses, meta-analyses, and trial sequential analyses of the effects of supplementation with beta-carotene, vitamin A, and vitamin E singly or in different combinations on all-cause mortality: do we have evidence for lack of harm? PLoS One. 2013; 8:e74558. 10.1371/journal.pone.0074558. 24040282 PMC3765487

[15] Omenn GS , Goodman GE , Thornquist MD , Balmes J , Cullen MR , Glass A , Keogh JP , Meyskens FL , Valanis B , Williams JH , Barnhart S , Hammar S . Effects of a combination of beta carotene and vitamin A on lung cancer and cardiovascular disease. N Engl J Med. 1996; 334:1150–5. 10.1056/nejm199605023341802. 8602180

[16] Goodman GE , Thornquist MD , Balmes J , Cullen MR , Meyskens FL Jr , Omenn GS , Valanis B , Williams JH Jr . The Beta-Carotene and Retinol Efficacy Trial: incidence of lung cancer and cardiovascular disease mortality during 6-year follow-up after stopping beta-carotene and retinol supplements. J Natl Cancer Inst. 2004; 96:1743–50. 10.1093/jnci/djh320. 15572756

[17] Wright ME , Virtamo J , Hartman AM , Pietinen P , Edwards BK , Taylor PR , Huttunen JK , Albanes D . Effects of alpha-tocopherol and beta-carotene supplementation on upper aerodigestive tract cancers in a large, randomized controlled trial. Cancer. 2007; 109:891–8. 10.1002/cncr.22482. 17265529

[18] Sayin VI , Ibrahim MX , Larsson E , Nilsson JA , Lindahl P , Bergo MO . Antioxidants accelerate lung cancer progression in mice. Sci Transl Med. 2014; 6:221ra15. 10.1126/scitranslmed.3007653. 24477002

[19] Piskounova E , Agathocleous M , Murphy MM , Hu Z , Huddlestun SE , Zhao Z , Leitch AM , Johnson TM , DeBerardinis RJ , Morrison SJ . Oxidative stress inhibits distant metastasis by human melanoma cells. Nature. 2015; 527:186–91. 10.1038/nature15726. 26466563 PMC4644103

[20] Blagosklonny MV . Prevention of cancer by inhibiting aging. Cancer Biol Ther. 2008; 7:1520–4. 10.4161/cbt.7.10.6663. 18769112

[21] Blagosklonny MV . Validation of anti-aging drugs by treating age-related diseases. Aging (Albany NY). 2009; 1:281–8. 10.18632/aging.100034. 20157517 PMC2806014

[22] Blagosklonny MV . Prospective treatment of age-related diseases by slowing down aging. Am J Pathol. 2012; 181:1142–6. 10.1016/j.ajpath.2012.06.024. 22841821

[23] Burch JB , Augustine AD , Frieden LA , Hadley E , Howcroft TK , Johnson R , Khalsa PS , Kohanski RA , Li XL , Macchiarini F , Niederehe G , Oh YS , Pawlyk AC , et al. Advances in geroscience: impact on healthspan and chronic disease. J Gerontol A Biol Sci Med Sci. 2014; 69:S1–3. 10.1093/gerona/glu041. 24833579 PMC4036419

[24] Justice J , Miller JD , Newman JC , Hashmi SK , Halter J , Austad SN , Barzilai N , Kirkland JL . Frameworks for Proof-of-Concept Clinical Trials of Interventions That Target Fundamental Aging Processes. J Gerontol A Biol Sci Med Sci. 2016; 71:1415–23. 10.1093/gerona/glw126. 27535966 PMC5055651

[25] Kennedy BK , Berger SL , Brunet A , Campisi J , Cuervo AM , Epel ES , Franceschi C , Lithgow GJ , Morimoto RI , Pessin JE , Rando TA , Richardson A , Schadt EE , et al. Geroscience: linking aging to chronic disease. Cell. 2014; 159:709–13. 10.1016/j.cell.2014.10.039. 25417146 PMC4852871

[26] Kaeberlein M . Translational geroscience: A new paradigm for 21(st) century medicine. Transl Med Aging. 2017; 1:1–4. 10.1016/j.tma.2017.09.004. 32219192 PMC7098696

[27] Kaeberlein M . How healthy is the healthspan concept? Geroscience. 2018; 40:361–64. 10.1007/s11357-018-0036-9. 30084059 PMC6136295

[28] Franceschi C , Passarino G , Mari D , Monti D . Centenarians as a 21st century healthy aging model: A legacy of humanity and the need for a world-wide consortium (WWC100+). Mech Ageing Dev. 2017; 165:55–58. 10.1016/j.mad.2017.06.002. 28651996

[29] Omodei D , Fontana L . Calorie restriction and prevention of age-associated chronic disease. FEBS Lett. 2011; 585:1537–42. 10.1016/j.febslet.2011.03.015. 21402069 PMC3439843

[30] Blagosklonny MV . Once again on rapamycin-induced insulin resistance and longevity: despite of or owing to. Aging (Albany NY). 2012; 4:350–8. 10.18632/aging.100461. 22683661 PMC3384435

[31] Bär C , Huber N , Beier F , Blasco MA . Therapeutic effect of androgen therapy in a mouse model of aplastic anemia produced by short telomeres. Haematologica. 2015; 100:1267–74. 10.3324/haematol.2015.129239. 26206796 PMC4591758

[32] Mattison JA , Colman RJ , Beasley TM , Allison DB , Kemnitz JW , Roth GS , Ingram DK , Weindruch R , de Cabo R , Anderson RM . Caloric restriction improves health and survival of rhesus monkeys. Nat Commun. 2017; 8:14063. 10.1038/ncomms14063. 28094793 PMC5247583

[33] Pifferi F , Terrien J , Perret M , Epelbaum J , Blanc S , Picq JL , Dhenain M , Aujard F . Promoting healthspan and lifespan with caloric restriction in primates. Commun Biol. 2019; 2:107. 10.1038/s42003-019-0348-z. 30911682 PMC6420603

[34] Shanley DP , Kirkwood TB . Caloric restriction does not enhance longevity in all species and is unlikely to do so in humans. Biogerontology. 2006; 7:165–8. 10.1007/s10522-006-9006-1. 16858629

[35] Pearson KJ , Baur JA , Lewis KN , Peshkin L , Price NL , Labinskyy N , Swindell WR , Kamara D , Minor RK , Perez E , Jamieson HA , Zhang Y , Dunn SR , et al. Resveratrol delays age-related deterioration and mimics transcriptional aspects of dietary restriction without extending life span. Cell Metab. 2008; 8:157–68. 10.1016/j.cmet.2008.06.011. 18599363 PMC2538685

[36] Miller RA , Harrison DE , Astle CM , Baur JA , Boyd AR , de Cabo R , Fernandez E , Flurkey K , Javors MA , Nelson JF , Orihuela CJ , Pletcher S , Sharp ZD , et al. Rapamycin, but not resveratrol or simvastatin, extends life span of genetically heterogeneous mice. J Gerontol A Biol Sci Med Sci. 2011; 66:191–201. 10.1093/gerona/glq178. 20974732 PMC3021372

[37] Strong R , Miller RA , Astle CM , Baur JA , de Cabo R , Fernandez E , Guo W , Javors M , Kirkland JL , Nelson JF , Sinclair DA , Teter B , Williams D , et al. Evaluation of resveratrol, green tea extract, curcumin, oxaloacetic acid, and medium-chain triglyceride oil on life span of genetically heterogeneous mice. J Gerontol A Biol Sci Med Sci. 2013; 68:6–16. 10.1093/gerona/gls070. 22451473 PMC3598361

[38] da Luz PL , Tanaka L , Brum PC , Dourado PM , Favarato D , Krieger JE , Laurindo FR . Red wine and equivalent oral pharmacological doses of resveratrol delay vascular aging but do not extend life span in rats. Atherosclerosis. 2012; 224:136–42. 10.1016/j.atherosclerosis.2012.06.007. 22818625

[39] Baur JA , Pearson KJ , Price NL , Jamieson HA , Lerin C , Kalra A , Prabhu VV , Allard JS , Lopez-Lluch G , Lewis K , Pistell PJ , Poosala S , Becker KG , et al. Resveratrol improves health and survival of mice on a high-calorie diet. Nature. 2006; 444:337–42. 10.1038/nature05354. 17086191 PMC4990206

[40] Chen C , Liu Y , Liu Y , Zheng P . mTOR regulation and therapeutic rejuvenation of aging hematopoietic stem cells. Sci Signal. 2009; 2:ra75. 10.1126/scisignal.2000559. 19934433 PMC4020596

[41] Harrison DE , Strong R , Sharp ZD , Nelson JF , Astle CM , Flurkey K , Nadon NL , Wilkinson JE , Frenkel K , Carter CS , Pahor M , Javors MA , Fernandez E , et al. Rapamycin fed late in life extends lifespan in genetically heterogeneous mice. Nature. 2009; 460:392–5. 10.1038/nature08221. 19587680 PMC2786175

[42] Anisimov VN , Zabezhinski MA , Popovich IG , Piskunova TS , Semenchenko AV , Tyndyk ML , Yurova MN , Antoch MP , Blagosklonny MV . Rapamycin extends maximal lifespan in cancer-prone mice. Am J Pathol. 2010; 176:2092–7. 10.2353/ajpath.2010.091050. 20363920 PMC2861075

[43] Anisimov VN , Zabezhinski MA , Popovich IG , Piskunova TS , Semenchenko AV , Tyndyk ML , Yurova MN , Rosenfeld SV , Blagosklonny MV . Rapamycin increases lifespan and inhibits spontaneous tumorigenesis in inbred female mice. Cell Cycle. 2011; 10:4230–6. 10.4161/cc.10.24.18486. 22107964

[44] Comas M , Toshkov I , Kuropatwinski KK , Chernova OB , Polinsky A , Blagosklonny MV , Gudkov AV , Antoch MP . New nanoformulation of rapamycin Rapatar extends lifespan in homozygous p53-/- mice by delaying carcinogenesis. Aging (Albany NY). 2012; 4:715–22. 10.18632/aging.100496. 23117593 PMC3517942

[45] Komarova EA , Antoch MP , Novototskaya LR , Chernova OB , Paszkiewicz G , Leontieva OV , Blagosklonny MV , Gudkov AV . Rapamycin extends lifespan and delays tumorigenesis in heterozygous p53+/- mice. Aging (Albany NY). 2012; 4:709–14. 10.18632/aging.100498. 23123616 PMC3517941

[46] Ramos FJ , Chen SC , Garelick MG , Dai DF , Liao CY , Schreiber KH , MacKay VL , An EH , Strong R , Ladiges WC , Rabinovitch PS , Kaeberlein M , Kennedy BK . Rapamycin reverses elevated mTORC1 signaling in lamin A/C-deficient mice, rescues cardiac and skeletal muscle function, and extends survival. Sci Transl Med. 2012; 4:144ra03. 10.1126/scitranslmed.3003802. 22837538 PMC3613228

[47] Wilkinson JE , Burmeister L , Brooks SV , Chan CC , Friedline S , Harrison DE , Hejtmancik JF , Nadon N , Strong R , Wood LK , Woodward MA , Miller RA . Rapamycin slows aging in mice. Aging Cell. 2012; 11:675–82. 10.1111/j.1474-9726.2012.00832.x. 22587563 PMC3434687

[48] Fang Y , Westbrook R , Hill C , Boparai RK , Arum O , Spong A , Wang F , Javors MA , Chen J , Sun LY , Bartke A . Duration of rapamycin treatment has differential effects on metabolism in mice. Cell Metab. 2013; 17:456–62. 10.1016/j.cmet.2013.02.008. 23473038 PMC3658445

[49] Flynn JM , O’Leary MN , Zambataro CA , Academia EC , Presley MP , Garrett BJ , Zykovich A , Mooney SD , Strong R , Rosen CJ , Kapahi P , Nelson MD , Kennedy BK , et al. Late-life rapamycin treatment reverses age-related heart dysfunction. Aging Cell. 2013; 12:851–62. 10.1111/acel.12109. 23734717 PMC4098908

[50] Johnson SC , Yanos ME , Kayser EB , Quintana A , Sangesland M , Castanza A , Uhde L , Hui J , Wall VZ , Gagnidze A , Oh K , Wasko BM , Ramos FJ , et al. mTOR inhibition alleviates mitochondrial disease in a mouse model of Leigh syndrome. Science. 2013; 342:1524–8. 10.1126/science.1244360. 24231806 PMC4055856

[51] Livi CB , Hardman RL , Christy BA , Dodds SG , Jones D , Williams C , Strong R , Bokov A , Javors MA , Ikeno Y , Hubbard G , Hasty P , Sharp ZD . Rapamycin extends life span of Rb1+/- mice by inhibiting neuroendocrine tumors. Aging (Albany NY). 2013; 5:100–10. 10.18632/aging.100533. 23454836 PMC3616197

[52] Neff F , Flores-Dominguez D , Ryan DP , Horsch M , Schröder S , Adler T , Afonso LC , Aguilar-Pimentel JA , Becker L , Garrett L , Hans W , Hettich MM , Holtmeier R , et al. Rapamycin extends murine lifespan but has limited effects on aging. J Clin Invest. 2013; 123:3272–91. 10.1172/jci67674. 23863708 PMC3726163

[53] Fok WC , Chen Y , Bokov A , Zhang Y , Salmon AB , Diaz V , Javors M , Wood WH 3rd , Zhang Y , Becker KG , Pérez VI , Richardson A . Mice fed rapamycin have an increase in lifespan associated with major changes in the liver transcriptome. PLoS One. 2014; 9:e83988. 10.1371/journal.pone.0083988. 24409289 PMC3883653

[54] Hasty P , Livi CB , Dodds SG , Jones D , Strong R , Javors M , Fischer KE , Sloane L , Murthy K , Hubbard G , Sun L , Hurez V , Curiel TJ , et al. eRapa restores a normal life span in a FAP mouse model. Cancer Prev Res (Phila). 2014; 7:169–78. https://doi.org/10.1158/1940-6207.capr-13-0299. 24282255 10.1158/1940-6207.CAPR-13-0299 PMC4058993

[55] Khapre RV , Kondratova AA , Patel S , Dubrovsky Y , Wrobel M , Antoch MP , Kondratov RV . BMAL1-dependent regulation of the mTOR signaling pathway delays aging. Aging (Albany NY). 2014; 6:48–57. 10.18632/aging.100633. 24481314 PMC3927809

[56] Leontieva OV , Paszkiewicz GM , Blagosklonny MV . Weekly administration of rapamycin improves survival and biomarkers in obese male mice on high-fat diet. Aging Cell. 2014; 13:616–22. https://doi.org/10.1111/acel.12211. 24655348 10.1111/acel.12211 PMC4326934

[57] Miller RA , Harrison DE , Astle CM , Fernandez E , Flurkey K , Han M , Javors MA , Li X , Nadon NL , Nelson JF , Pletcher S , Salmon AB , Sharp ZD , et al. Rapamycin-mediated lifespan increase in mice is dose and sex dependent and metabolically distinct from dietary restriction. Aging Cell. 2014; 13:468–77. 10.1111/acel.12194. 24341993 PMC4032600

[58] Popovich IG , Anisimov VN , Zabezhinski MA , Semenchenko AV , Tyndyk ML , Yurova MN , Blagosklonny MV . Lifespan extension and cancer prevention in HER-2/neu transgenic mice treated with low intermittent doses of rapamycin. Cancer Biol Ther. 2014; 15:586–92. 10.4161/cbt.28164. 24556924 PMC4026081

[59] Zhang Y , Bokov A , Gelfond J , Soto V , Ikeno Y , Hubbard G , Diaz V , Sloane L , Maslin K , Treaster S , Réndon S , van Remmen H , Ward W , et al. Rapamycin extends life and health in C57BL/6 mice. J Gerontol A Biol Sci Med Sci. 2014; 69:119–30. 10.1093/gerona/glt056. 23682161 PMC4038246

[60] Hurez V , Dao V , Liu A , Pandeswara S , Gelfond J , Sun L , Bergman M , Orihuela CJ , Galvan V , Padrón Á , Drerup J , Liu Y , Hasty P , et al. Chronic mTOR inhibition in mice with rapamycin alters T, B, myeloid, and innate lymphoid cells and gut flora and prolongs life of immune-deficient mice. Aging Cell. 2015; 14:945–56. 10.1111/acel.12380. 26315673 PMC4693453

[61] Johnson SC , Yanos ME , Bitto A , Castanza A , Gagnidze A , Gonzalez B , Gupta K , Hui J , Jarvie C , Johnson BM , Letexier N , McCanta L , Sangesland M , et al. Dose-dependent effects of mTOR inhibition on weight and mitochondrial disease in mice. Front Genet. 2015; 6:247. 10.3389/fgene.2015.00247. 26257774 PMC4510413

[62] Arriola Apelo SI , Pumper CP , Baar EL , Cummings NE , Lamming DW . Intermittent Administration of Rapamycin Extends the Life Span of Female C57BL/6J Mice. J Gerontol A Biol Sci Med Sci. 2016; 71:876–81. 10.1093/gerona/glw064. 27091134 PMC4906329

[63] Bitto A , Ito TK , Pineda VV , LeTexier NJ , Huang HZ , Sutlief E , Tung H , Vizzini N , Chen B , Smith K , Meza D , Yajima M , Beyer RP , et al. Transient rapamycin treatment can increase lifespan and healthspan in middle-aged mice. Elife. 2016; 5:e16351. 10.7554/elife.16351. 27549339 PMC4996648

[64] Liao CY , Anderson SS , Chicoine NH , Mayfield JR , Academia EC , Wilson JA , Pongkietisak C , Thompson MA , Lagmay EP , Miller DM , Hsu YM , McCormick MA , O‘Leary MN , et al. Rapamycin Reverses Metabolic Deficits in Lamin A/C-Deficient Mice. Cell Rep. 2016; 17:2542–52. 10.1016/j.celrep.2016.10.040. 27926859 PMC6594831

[65] Felici R , Buonvicino D , Muzzi M , Cavone L , Guasti D , Lapucci A , Pratesi S , De Cesaris F , Luceri F , Chiarugi A . Post onset, oral rapamycin treatment delays development of mitochondrial encephalopathy only at supramaximal doses. Neuropharmacology. 2017; 117:74–84. 10.1016/j.neuropharm.2017.01.039. 28161373

[66] Siegmund SE , Yang H , Sharma R , Javors M , Skinner O , Mootha V , Hirano M , Schon EA . Low-dose rapamycin extends lifespan in a mouse model of mtDNA depletion syndrome. Hum Mol Genet. 2017; 26:4588–605. 10.1093/hmg/ddx341. 28973153 PMC5886265

[67] Bielas J , Herbst A , Widjaja K , Hui J , Aiken JM , McKenzie D , Miller RA , Brooks SV , Wanagat J . Long term rapamycin treatment improves mitochondrial DNA quality in aging mice. Exp Gerontol. 2018; 106:125–31. 10.1016/j.exger.2018.02.021. 29486228 PMC5911406

[68] Ferrara-Romeo I , Martinez P , Saraswati S , Whittemore K , Graña-Castro O , Thelma Poluha L , Serrano R , Hernandez-Encinas E , Blanco-Aparicio C , Maria Flores J , Blasco MA . The mTOR pathway is necessary for survival of mice with short telomeres. Nat Commun. 2020; 11:1168. 10.1038/s41467-020-14962-1. 32127537 PMC7054554

[69] Parihar M , Dodds SG , Hubbard G , Javors MA , Strong R , Hasty P , Sharp ZD . Rapamycin Extends Life Span in Apc(Min/+) Colon Cancer FAP Model. Clin Colorectal Cancer. 2020 9 15. 10.1016/j.clcc.2020.08.006. [Epub ahead of print]. 33132009 PMC7956131

[70] Strong R , Miller RA , Bogue M , Fernandez E , Javors MA , Libert S , Marinez PA , Murphy MP , Musi N , Nelson JF , Petrascheck M , Reifsnyder P , Richardson A , et al. Rapamycin-mediated mouse lifespan extension: Late-life dosage regimes with sex-specific effects. Aging Cell. 2020; 19:e13269. 10.1111/acel.13269. 33145977 PMC7681050

[71] Spindler SR , Mote PL , Flegal JM , Teter B . Influence on longevity of blueberry, cinnamon, green and black tea, pomegranate, sesame, curcumin, morin, pycnogenol, quercetin, and taxifolin fed iso-calorically to long-lived, F1 hybrid mice. Rejuvenation Res. 2013; 16:143–51. 10.1089/rej.2012.1386. 23432089

[72] Jones E , Hughes RE . Quercetin, flavonoids and the life-span of mice. Exp Gerontol. 1982; 17:213–7. 10.1016/0531-5565(82)90027-4. 7140862

[73] Xu M , Pirtskhalava T , Farr JN , Weigand BM , Palmer AK , Weivoda MM , Inman CL , Ogrodnik MB , Hachfeld CM , Fraser DG , Onken JL , Johnson KO , Verzosa GC , et al. Senolytics improve physical function and increase lifespan in old age. Nat Med. 2018; 24:1246–56. 10.1038/s41591-018-0092-9. 29988130 PMC6082705

[74] Filfan M , Olaru A , Udristoiu I , Margaritescu C , Petcu E , Hermann DM , Popa-Wagner A . Long-term treatment with spermidine increases health span of middle-aged Sprague-Dawley male rats. Geroscience. 2020; 42:937–49. 10.1007/s11357-020-00173-5. 32285289 PMC7287009

[75] Eisenberg T , Abdellatif M , Schroeder S , Primessnig U , Stekovic S , Pendl T , Harger A , Schipke J , Zimmermann A , Schmidt A , Tong M , Ruckenstuhl C , Dammbrueck C , et al. Cardioprotection and lifespan extension by the natural polyamine spermidine. Nat Med. 2016; 22:1428–38. 10.1038/nm.4222. 27841876 PMC5806691

[76] Zhang H , Ryu D , Wu Y , Gariani K , Wang X , Luan P , D‘Amico D , Ropelle ER , Lutolf MP , Aebersold R , Schoonjans K , Menzies KJ , Auwerx J . NAD(+) repletion improves mitochondrial and stem cell function and enhances life span in mice. Science. 2016; 352:1436–43. 10.1126/science.aaf2693. 27127236

[77] Mitchell SJ , Bernier M , Aon MA , Cortassa S , Kim EY , Fang EF , Palacios HH , Ali A , Navas-Enamorado I , Di Francesco A , Kaiser TA , Waltz TB , Zhang N , et al. Nicotinamide Improves Aspects of Healthspan, but Not Lifespan, in Mice. Cell Metab. 2018; 27:667–76.e4. 10.1016/j.cmet.2018.02.001. 29514072 PMC5854409

[78] Fang EF , Kassahun H , Croteau DL , Scheibye-Knudsen M , Marosi K , Lu H , Shamanna RA , Kalyanasundaram S , Bollineni RC , Wilson MA , Iser WB , Wollman BN , Morevati M , et al. NAD(+) Replenishment Improves Lifespan and Healthspan in Ataxia Telangiectasia Models via Mitophagy and DNA Repair. Cell Metab. 2016; 24:566–81. 10.1016/j.cmet.2016.09.004. 27732836 PMC5777858

[79] Lee CF , Caudal A , Abell L , Nagana Gowda GA , Tian R . Targeting NAD(+) Metabolism as Interventions for Mitochondrial Disease. Sci Rep. 2019; 9:3073. 10.1038/s41598-019-39419-4. 30816177 PMC6395802

[80] Ackerman D , Gems D . The mystery of C. elegans aging: an emerging role for fat. Distant parallels between C. elegans aging and metabolic syndrome? Bioessays. 2012; 34:466–71. 10.1002/bies.201100189. 22371137

[81] Dang Y , An Y , He J , Huang B , Zhu J , Gao M , Zhang S , Wang X , Yang B , Xie Z . Berberine ameliorates cellular senescence and extends the lifespan of mice via regulating p16 and cyclin protein expression. Aging Cell. 2020; 19:e13060. 10.1111/acel.13060. 31773901 PMC6974710

[82] Zhao H , Halicka HD , Li J , Darzynkiewicz Z . Berberine suppresses gero-conversion from cell cycle arrest to senescence. Aging (Albany NY). 2013; 5:623–36. 10.18632/aging.100593. 23974852 PMC3796215

[83] Yousefzadeh MJ , Zhu Y , McGowan SJ , Angelini L , Fuhrmann-Stroissnigg H , Xu M , Ling YY , Melos KI , Pirtskhalava T , Inman CL , McGuckian C , Wade EA , Kato JI , et al. Fisetin is a senotherapeutic that extends health and lifespan. EBioMedicine. 2018; 36:18–28. 10.1016/j.ebiom.2018.09.015. 30279143 PMC6197652

[84] Anisimov VN , Piskunova TS , Popovich IG , Zabezhinski MA , Tyndyk ML , Egormin PA , Yurova MV , Rosenfeld SV , Semenchenko AV , Kovalenko IG , Poroshina TE , Berstein LM . Gender differences in metformin effect on aging, life span and spontaneous tumorigenesis in 129/Sv mice. Aging (Albany NY). 2010; 2:945–58. 10.18632/aging.100245. 21164223 PMC3034183

[85] Anisimov VN , Berstein LM , Egormin PA , Piskunova TS , Popovich IG , Zabezhinski MA , Tyndyk ML , Yurova MV , Kovalenko IG , Poroshina TE , Semenchenko AV . Metformin slows down aging and extends life span of female SHR mice. Cell Cycle. 2008; 7:2769–73. 10.4161/cc.7.17.6625. 18728386

[86] Anisimov VN , Berstein LM , Popovich IG , Zabezhinski MA , Egormin PA , Piskunova TS , Semenchenko AV , Tyndyk ML , Yurova MN , Kovalenko IG , Poroshina TE . If started early in life, metformin treatment increases life span and postpones tumors in female SHR mice. Aging (Albany NY). 2011; 3:148–57. 10.18632/aging.100273. 21386129 PMC3082009

[87] Palliyaguru DL , Minor RK , Mitchell SJ , Palacios HH , Licata JJ , Ward TM , Abulwerdi G , Elliott P , Westphal C , Ellis JL , Sinclair DA , Price NL , Bernier M , de Cabo R . Combining a High Dose of Metformin With the SIRT1 Activator, SRT1720, Reduces Life Span in Aged Mice Fed a High-Fat Diet. J Gerontol A Biol Sci Med Sci. 2020; 75:2037–41. 10.1093/gerona/glaa148. 32556267 PMC7750506

[88] Anisimov VN , Popovich IG , Zabezhinski MA , Egormin PA , Yurova MN , Semenchenko AV , Tyndyk ML , Panchenko AV , Trashkov AP , Vasiliev AG , Khaitsev NV . Sex differences in aging, life span and spontaneous tumorigenesis in 129/Sv mice neonatally exposed to metformin. Cell Cycle. 2015; 14:46–55. 10.4161/15384101.2014.973308. 25483062 PMC4353070

[89] Martin-Montalvo A , Mercken EM , Mitchell SJ , Palacios HH , Mote PL , Scheibye-Knudsen M , Gomes AP , Ward TM , Minor RK , Blouin MJ , Schwab M , Pollak M , Zhang Y , et al. Metformin improves healthspan and lifespan in mice. Nat Commun. 2013; 4:2192. 10.1038/ncomms3192. 23900241 PMC3736576

[90] Strong R , Miller RA , Antebi A , Astle CM , Bogue M , Denzel MS , Fernandez E , Flurkey K , Hamilton KL , Lamming DW , Javors MA , de Magalhães J , Martinez PA , et al. Longer lifespan in male mice treated with a weakly estrogenic agonist, an antioxidant, an alpha-glucosidase inhibitor or a Nrf2-inducer. Aging Cell. 2016; 15:872–84. 10.1111/acel.12496. 27312235 PMC5013015

[91] Anisimov VN , Berstein LM , Egormin PA , Piskunova TS , Popovich IG , Zabezhinski MA , Kovalenko IG , Poroshina TE , Semenchenko AV , Provinciali M , Re F , Franceschi C . Effect of metformin on life span and on the development of spontaneous mammary tumors in HER-2/neu transgenic mice. Exp Gerontol. 2005; 40:685–93. 10.1016/j.exger.2005.07.007. 16125352

[92] Anisimov VN , Egormin PA , Piskunova TS , Popovich IG , Tyndyk ML , Yurova MN , Zabezhinski MA , Anikin IV , Karkach AS , Romanyukha AA . Metformin extends life span of HER-2/neu transgenic mice and in combination with melatonin inhibits growth of transplantable tumors *in vivo* . Cell Cycle. 2010; 9:188–97. 10.4161/cc.9.1.10407. 20016287

[93] Harrison DE , Strong R , Allison DB , Ames BN , Astle CM , Atamna H , Fernandez E , Flurkey K , Javors MA , Nadon NL , Nelson JF , Pletcher S , Simpkins JW , et al. Acarbose, 17-alpha-estradiol, and nordihydroguaiaretic acid extend mouse lifespan preferentially in males. Aging Cell. 2014; 13:273–82. 10.1111/acel.12170. 24245565 PMC3954939

[94] Harrison DE , Strong R , Alavez S , Astle CM , DiGiovanni J , Fernandez E , Flurkey K , Garratt M , Gelfond JAL , Javors MA , Levi M , Lithgow GJ , Macchiarini F , et al. Acarbose improves health and lifespan in aging HET3 mice. Aging Cell. 2019; 18:e12898. 10.1111/acel.12898. 30688027 PMC6413665

[95] Dodds SG , Parihar M , Javors M , Nie J , Musi N , Dave Sharp Z , Hasty P . Acarbose improved survival for Apc(+/Min) mice. Aging Cell. 2020; 19:e13088. 10.1111/acel.13088. 31903726 PMC6996958

[96] Blagosklonny MV . The mystery of the ketogenic diet: benevolent pseudo-diabetes. Cell Cycle. 2019; 18:2157–63. 10.1080/15384101.2019.1644765. 31368400 PMC6738531

[97] Ferder L , Inserra F , Romano L , Ercole L , Pszenny V . Effects of angiotensin-converting enzyme inhibition on mitochondrial number in the aging mouse. Am J Physiol. 1993; 265:C15–8. 10.1152/ajpcell.1993.265.1.c15. 8338123

[98] Basso N , Cini R , Pietrelli A , Ferder L , Terragno NA , Inserra F . Protective effect of long-term angiotensin II inhibition. Am J Physiol Heart Circ Physiol. 2007; 293:H1351–8. 10.1152/ajpheart.00393.2007. 17557916

[99] Santos EL , de Picoli Souza K , da Silva ED , Batista EC , Martins PJ , D‘Almeida V , Pesquero JB . Long term treatment with ACE inhibitor enalapril decreases body weight gain and increases life span in rats. Biochem Pharmacol. 2009; 78:951–8. 10.1016/j.bcp.2009.06.018. 19549507

[100] Spindler SR , Mote PL , Flegal JM . Combined statin and angiotensin-converting enzyme (ACE) inhibitor treatment increases the lifespan of long-lived F1 male mice. Age (Dordr). 2016; 38:379–91. 10.1007/s11357-016-9948-4. 27590905 PMC5266223

[101] Blagosklonny MV . An anti-aging drug today: from senescence-promoting genes to anti-aging pill. Drug Discov Today. 2007; 12:218–24. 10.1016/j.drudis.2007.01.004. 17331886

[102] Demidenko ZN , Blagosklonny MV . Growth stimulation leads to cellular senescence when the cell cycle is blocked. Cell Cycle. 2008; 7:3355–61. 10.4161/cc.7.21.6919. 18948731

[103] Demidenko ZN , Shtutman M , Blagosklonny MV . Pharmacologic inhibition of MEK and PI-3K converges on the mTOR/S6 pathway to decelerate cellular senescence. Cell Cycle. 2009; 8:1896–900. 10.4161/cc.8.12.8809. 19478560

[104] Leontieva OV , Blagosklonny MV . Gerosuppression by pan-mTOR inhibitors. Aging (Albany NY). 2016; 8:3535–51. 10.18632/aging.101155. 28077803 PMC5270685

[105] Blagosklonny MV . Rapamycin and quasi-programmed aging: four years later. Cell Cycle. 2010; 9:1859–62. 10.4161/cc.9.10.11872. 20436272

[106] Athar M , Back JH , Kopelovich L , Bickers DR , Kim AL . Multiple molecular targets of resveratrol: Anti-carcinogenic mechanisms. Arch Biochem Biophys. 2009; 486:95–102. 10.1016/j.abb.2009.01.018. 19514131 PMC2749321

[107] Armour SM , Baur JA , Hsieh SN , Land-Bracha A , Thomas SM , Sinclair DA . Inhibition of mammalian S6 kinase by resveratrol suppresses autophagy. Aging (Albany NY). 2009; 1:515–28. 10.18632/aging.100056. 20157535 PMC2806030

[108] Demidenko ZN , Blagosklonny MV . At concentrations that inhibit mTOR, resveratrol suppresses cellular senescence. Cell Cycle. 2009; 8:1901–4. 10.4161/cc.8.12.8810. 19471118

[109] Lee JH , Wendorff TJ , Berger JM . Resveratrol: A novel type of topoisomerase II inhibitor. J Biol Chem. 2017; 292:21011–22. 10.1074/jbc.m117.810580. 29074616 PMC5743075

[110] Campbell JM , Bellman SM , Stephenson MD , Lisy K . Metformin reduces all-cause mortality and diseases of ageing independent of its effect on diabetes control: A systematic review and meta-analysis. Ageing Res Rev. 2017; 40:31–44. 10.1016/j.arr.2017.08.003. 28802803

[111] Han Y , Xie H , Liu Y , Gao P , Yang X , Shen Z . Effect of metformin on all-cause and cardiovascular mortality in patients with coronary artery diseases: a systematic review and an updated meta-analysis. Cardiovasc Diabetol. 2019; 18:96. 10.1186/s12933-019-0900-7. 31362743 PMC6668189

[112] Hu Y , Lei M , Ke G , Huang X , Peng X , Zhong L , Fu P . Metformin Use and Risk of All-Cause Mortality and Cardiovascular Events in Patients With Chronic Kidney Disease-A Systematic Review and Meta-Analysis. Front Endocrinol (Lausanne). 2020; 11:559446. 10.3389/fendo.2020.559446. 33117278 PMC7575818

[113] Blagosklonny MV . Koschei the immortal and anti-aging drugs. Cell Death Dis. 2014; 5:e1552. 10.1038/cddis.2014.520. 25476900 PMC4649836

[114] Cheng J , Zhang W , Zhang X , Han F , Li X , He X , Li Q , Chen J . Effect of angiotensin-converting enzyme inhibitors and angiotensin II receptor blockers on all-cause mortality, cardiovascular deaths, and cardiovascular events in patients with diabetes mellitus: a meta-analysis. JAMA Intern Med. 2014; 174:773–85. 10.1001/jamainternmed.2014.348. 24687000

[115] Lv X , Zhang Y , Niu Y , Song Q , Zhao Q . Comparison of angiotensin-converting enzyme inhibitors and angiotensin II receptor blockers on cardiovascular outcomes in hypertensive patients with type 2 diabetes mellitus: A PRISMA-compliant systematic review and meta-analysis. Medicine (Baltimore). 2018; 97:e0256. 10.1097/md.0000000000010256. 29642146 PMC5908573

[116] Tai C , Gan T , Zou L , Sun Y , Zhang Y , Chen W , Li J , Zhang J , Xu Y , Lu H , Xu D . Effect of angiotensin-converting enzyme inhibitors and angiotensin II receptor blockers on cardiovascular events in patients with heart failure: a meta-analysis of randomized controlled trials. BMC Cardiovasc Disord. 2017; 17:257. 10.1186/s12872-017-0686-z. 28982370 PMC5629775

[117] Messerli FH , Bangalore S , Bavishi C , Rimoldi SF . Angiotensin-Converting Enzyme Inhibitors in Hypertension: To Use or Not to Use? J Am Coll Cardiol. 2018; 71:1474–82. 10.1016/j.jacc.2018.01.058. 29598869

[118] Benigni A , Corna D , Zoja C , Sonzogni A , Latini R , Salio M , Conti S , Rottoli D , Longaretti L , Cassis P , Morigi M , Coffman TM , Remuzzi G . Disruption of the Ang II type 1 receptor promotes longevity in mice. J Clin Invest. 2009; 119:524–30. 10.1172/jci36703. 19197138 PMC2648681

[119] Blagosklonny MV . Rapamycin, proliferation and geroconversion to senescence. Cell Cycle. 2018; 17:2655–65. 10.1080/15384101.2018.1554781. 30541374 PMC6343718

[120] Demidenko ZN , Korotchkina LG , Gudkov AV , Blagosklonny MV . Paradoxical suppression of cellular senescence by p53. Proc Natl Acad Sci U S A. 2010; 107:9660–4. 10.1073/pnas.1002298107. 20457898 PMC2906905

[121] Leontieva OV , Natarajan V , Demidenko ZN , Burdelya LG , Gudkov AV , Blagosklonny MV . Hypoxia suppresses conversion from proliferative arrest to cellular senescence. Proc Natl Acad Sci U S A. 2012; 109:13314–8. 10.1073/pnas.1205690109. 22847439 PMC3421205

[122] Leontieva OV , Demidenko ZN , Blagosklonny MV . S6K in geroconversion. Cell Cycle. 2013; 12:3249–52. 10.4161/cc.26248. 24036549 PMC3885635

[123] Castillo-Quan JI , Tain LS , Kinghorn KJ , Li L , Gronke S , Hinze Y , Blackwell TK , Bjedov I , Partridge L . A triple drug combination targeting components of the nutrient-sensing network maximizes longevity. Proc Natl Acad Sci U S A. 2019; 116:20817–19. 10.1073/pnas.1913212116. 31570569 PMC6800352

[124] Blagosklonny MV . How to save Medicare: the anti-aging remedy. Aging (Albany NY). 2012; 4:547–52. 10.18632/aging.100479. 22915707 PMC3461342

[125] Gems D . The aging-disease false dichotomy: understanding senescence as pathology. Front Genet. 2015; 6:212. 10.3389/fgene.2015.00212. 26136770 PMC4468941

[126] Barzilai N , Crandall JP , Kritchevsky SB , Espeland MA . Metformin as a Tool to Target Aging. Cell Metab. 2016; 23:1060–65. 10.1016/j.cmet.2016.05.011. 27304507 PMC5943638

[127] Sierra F . The Emergence of Geroscience as an Interdisciplinary Approach to the Enhancement of Health Span and Life Span. Cold Spring Harb Perspect Med. 2016; 6:a025163. 10.1101/cshperspect.a025163. 26931460 PMC4817738

[128] Kirkland JL , Tchkonia T . Clinical strategies and animal models for developing senolytic agents. Exp Gerontol. 2015; 68:19–25. 10.1016/j.exger.2014.10.012. 25446976 PMC4412760

[129] Hickson LJ , Langhi Prata LGP , Bobart SA , Evans TK , Giorgadze N , Hashmi SK , Herrmann SM , Jensen MD , Jia Q , Jordan KL , Kellogg TA , Khosla S , Koerber DM , et al. Senolytics decrease senescent cells in humans: Preliminary report from a clinical trial of Dasatinib plus Quercetin in individuals with diabetic kidney disease. EBioMedicine. 2019; 47:446–56. 10.1016/j.ebiom.2019.08.069. 31542391 PMC6796530

[130] Kirkland JL , Tchkonia T . Senolytic drugs: from discovery to translation. J Intern Med. 2020; 288:518–36. 10.1111/joim.13141. 32686219 PMC7405395

[131] Blagosklonny MV . Rapamycin for longevity: opinion article. Aging (Albany NY). 2019; 11:8048–67. 10.18632/aging.102355. 31586989 PMC6814615

